# The Unallotted Fund and Excessive Prescribing

**Published:** 1914-10-31

**Authors:** 


### LONDON PANEL COMMITTEE.

### The Unallotted Fund and Excessive Prescribing.

A number of important matters were considered at a meeting of the London Panel Committee on Tuesday last. It was reported that 1,150 practitioners on the panel in London had offered to undertake the medical treatment, free of charge, during the war, of necessitous dependants of Reservists and of insured persons unemployed because of the war. Dr. B. A. Richmond, the secretary of the Committee, remarked that as seventy practitioners had been called up for military duty, a very satisfactory proportion of panel practitioners had responded to the Committee's appeal.

### Panel Men and Pathological Laboratories.

The deputation appointed to express the views of panel practitioners to the Local Government Board with regard to the proposed establishment of pathological laboratories reported that it was very cordially received by Mr. Willis and Dr. Newsholme, but that, owing to the war, the proposed grants-in-aid must be withheld for the present. The officials of the Board agreed that the laboratories should be limited in number, so that they might be fully staffed with specialists and well equipped. The deputation urged the importance of the pathologist being in touch with individual practitioners, and it was understood that provision would be made for specimens to be taken when necessary by the pathologist himself. It appeared that the service would be a public health one, and the assistance of the laboratories—which would be established where possible in connection with hospitals—would be available for both insured and uninsured persons.

### The Unallotted Fund.

Some discussion took place over the assignment to practitioners of insured persons who have not chosen a doctor, and the crediting of additional capitation fees in respect of such persons. The London Insurance Committee, on October 22, decided to incorporate in the draft scheme agreed upon with the Panel Committee a proviso that in calculating the additional capitation fees no account should be taken of removals or persons changing their doctor at the end of the medical year, so that further capitation fees should be restricted to those persons who had not previously made a selection. In view of the fact that a practitioner did not receive payment during a particular quarter in respect of persons transferred to him during that quarter, the Service Sub-Committee advised the Panel Committee not to acquiesce in the modification.

Dr. R. V. Donnellan moved and Dr. H. H. Mills seconded an amendment that the suggestion of the Insurance Committee be accepted, and Dr. Cowie urged that the calculation of the unallotted funds must not be complicated by the introduction of persons who were in no sense unallotted. Dr. H. J. Cardale claimed that the scheme as originally approved by the Panel Committee did justice to the small-list practitioners, and would be an inducement to doctors to go on the panel. If transfers were excluded, and a doctor's old patients went back to him, the doctor would receive no share of the unallotted funds.

On a division the scheme, as amended by the Insurance Committee, was accepted by forty-one votes to eight.

### Alleged Cases of Excessive Prescribing.

The first cases came before the Committee in which allegations of excessive prescribing had been investigated and reported upon. Only one was dealt with by the meeting, and in that case the Committee expressed the opinion that the cost of the drugs and appliances ordered was excessive. The practitioner had 1,341 persons on his list, and the cost of 465 prescription forms issued by him in a certain period averaged 2s. 1d., the average cost per insured person being 8¾d. Eighty per cent. exceeded one shilling in value. Apart from too frequent repetition of a medicine, it appeared that the practitioner ordered larger quantities than were necessary. In defence, the practitioner urged that he could not be held responsible, nor refuse to prescribe, if a patient stated that a medicine had been consumed.

### The Analysis of Prescriptions.

The Panel Committee was asked to state whether it would continue to contribute £800 a year for the statistical analysis of prescriptions. In reply to Dr. Butler, who deprecated the expense as unnecessary, Dr. Coode Adams remarked that the drain on the drug fund was becoming so severe that the very existence of the Insurance Act was endangered. The chemists were extremely dissatisfied, and in London threatened not to accept the agreements next year. If panel practitioners wished the Insurance Act to continue they must deal strictly with over-prescribing, and the expenditure on the examination of prescriptions was necessary to that end. Dr. Richards hoped that members had read the Scottish report on this subject. This showed that in Scotland only one or two cities exceeded the 2s.

Dr. Donnellan mentioned that the total drug fund for 1914 has been expended at the end of September.

The Committee decided, with only two dissentients, to renew its contribution towards the examination of prescriptions.

